# PKCα-mediated phosphorylation of LSD1 is required for presynaptic plasticity and hippocampal learning and memory

**DOI:** 10.1038/s41598-017-05239-7

**Published:** 2017-07-07

**Authors:** Chae-Seok Lim, Hye Jin Nam, Jaehyun Lee, Dongha Kim, Ja Eun Choi, SukJae Joshua Kang, Somi Kim, Hyopil Kim, Chuljung Kwak, Kyu-Won Shim, Siyong Kim, Hyoung-Gon Ko, Ro Un Lee, Eun-Hae Jang, Juyoun Yoo, Jaehoon Shim, Md Ariful Islam, Yong-Seok Lee, Jae-Hyung Lee, Sung Hee Baek, Bong-Kiun Kaang

**Affiliations:** 1 Laboratory of Neurobiology, School of Biological Sciences, College of Natural Sciences, Seoul National University, Seoul, 08826 Korea; 2 Laboratory of Molecular and Cellular Genetics, School of Biological Sciences, College of Natural Sciences, Seoul National University, Seoul, 08826 Korea; 3 Department of Physiology, Biomedical Sciences, Seoul National University College of Medicine, Seoul, 03080 Korea; 4 Department of Life and Nanopharmaceutical Sciences, Department of Maxillofacial Biomedical Engineering, School of Dentistry, Kyung Hee University, Seoul, 02447 Korea

## Abstract

Lysine-specific demethylase 1 (LSD1) is a histone demethylase that participates in transcriptional repression or activation. Recent studies reported that LSD1 is involved in learning and memory. Although LSD1 phosphorylation by PKCα was implicated in circadian rhythmicity, the importance of LSD1 phosphorylation in learning and memory is unknown. In this study, we examined the roles of LSD1 in synaptic plasticity and memory using *Lsd1* ^SA/SA^ knock-in (KI) mice, in which a PKCα phosphorylation site is mutated. Interestingly, short-term and long-term contextual fear memory as well as spatial memory were impaired in *Lsd1* KI mice. In addition, short-term synaptic plasticity, such as paired pulse ratio and post-tetanic potentiation was impaired, whereas long-term synaptic plasticity, including long-term potentiation and long-term depression, was normal. Moreover, the frequency of miniature excitatory postsynaptic current was significantly increased, suggesting presynaptic dysfunction in *Lsd1* KI mice. Consistent with this, RNA-seq analysis using the hippocampus of *Lsd1* KI mice showed significant alterations in the expressions of presynaptic function-related genes. Intriguingly, LSD1n-SA mutant showed diminished binding to histone deacetylase 1 (HDAC1) compared to LSD1n-WT in SH-SY5Y cells. These results suggest that LSD1 is involved in the regulation of presynaptic gene expression and subsequently regulates the hippocampus-dependent memory in phosphorylation-dependent manner.

## Introduction

Lysine-specific demethylase 1 (LSD1, also known as KDM1A) is the first discovered histone lysine-specific demethylase and acts on mono- and di-methylated histone H3K4 or H3K9 via flavin adenine dinucleotide (FAD)-dependent amine oxidation reaction^1, 2^. LSD1 interacts with corepressor of REST (CoREST) and histone deacetylase 1 (HDAC1), resulting in participation in gene repression, and also interacts with androgen receptor (AR) to stimulate AR-dependent gene activation^3–5^. LSD1 plays critical roles in embryogenesis and tissue-specific differentiation as well as tumor cell growth^6–8^. Moreover, the interaction of LSD1 with adenylyl cyclase 3 (Adcy3) is necessary for stabilizing singular expression of an olfactory receptor (OR) by epigenetic modification of the selected OR gene^9^.

The epigenetic modification is important for synaptic plasticity and brain function^10, 11^. The modification of methylation on H3K9 by LSD1 is involved in changes in gene expression necessary for fear memory consolidation and is regulated by NMDA receptor-ERK signaling^12, 13^. Meanwhile, it was reported that inhibiting LSD1 using a specific inhibitor RN-1 immediately after novel object recognition (NOR) training impaired long-term memory^14^. In addition, a neuron-specific isoform of LSD1 (LSD1n), which results from an alternative splicing and has a histone H4K20 demethylase activity instead of H3K4 demethylase activity, was shown to be involved in learning and memory by regulating transcriptional elongation^15^. Importantly, *de novo* heterozygous missense mutations in the LSD1 (E403K, D580G, or Y785H) were found in patients with syndromic mental retardation^16, 17^. Recently, LSD1 phosphorylation by protein kinase C alpha (PKCα) was implicated in circadian rhythmicity, independent of its demethylase activity^18^. Perturbing the circadian system could also affect the memory of an animal^19, 20^. Mutant mice lacking an essential circadian clock-related gene, *Bmal1* exhibited deficits in hippocampus-dependent contextual fear and spatial memory as well as in LTP at the Schaffer-collateral (SC)-CA1 synapse^19, 20^. Therefore, it would be interesting to examine the implication/effect of LSD1 phosphorylation by PKCα in hippocampal learning and plasticity.

In this study, we demonstrated that LSD1 is involved in short-term synaptic plasticity and hippocampus-dependent learning and memory using the *Lsd1* ^SA/SA^ knock-in (KI) mouse expressing phosphorylation-defective LSD1, in which the serine at 112 residue of LSD1 is replaced with alanine^18^. Furthermore, *Lsd1* KI mice show altered expression in presynaptic plasticity-related genes, suggesting that LSD1 phosphorylation by PKCα is critical for presynaptic plasticity and memory.

## Results

### Hippocampus-dependent memory was impaired in *Lsd1* knock-in (KI) mice

**Figure 1:**
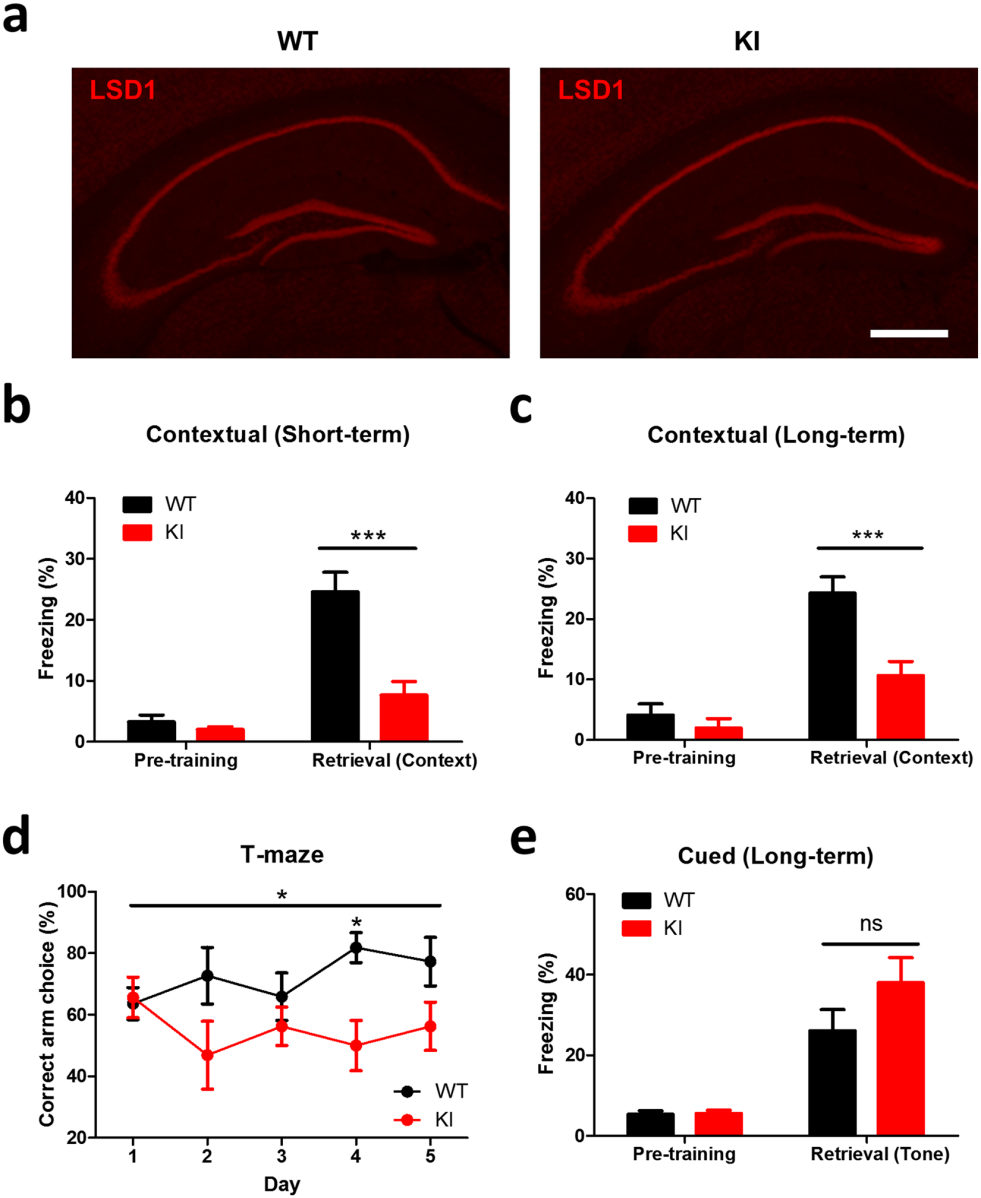
*Lsd1* knock-in (KI) mice show impaired hippocampus-dependent fear memory. (**a**) Representative immunohistochemical images (from 5 different animals for each genotype) showed no difference in LSD1 expression pattern in the hippocampus of WT and *Lsd1* KI mice. Scale bar: 500 μm. (**b**) Freezing levels of WT (black) and *Lsd1* KI (red) mice before (pretraining) and 1 h after (retrieval) contextual fear conditioning. *Lsd1* KI mice showed significantly reduced freezing levels during retrieval test compared to WT (WT: n = 7, KI: n = 6; two-way ANOVA, genotype x condition, *F*_1,22_ = 13.58, *p* < 0.01; effect of genotype, *F*_1,22_ = 18.21, *p* < 0.001; effect of condition, *F*_1,22_ = 40.07, *p* < 0.0001; Bonferroni posttests, ***p < 0.001). (**c**) Freezing levels of WT (black) and *Lsd1* KI (red) mice before (pretraining) and 24 h after (retrieval) contextual fear conditioning. *Lsd1* KI mice showed significantly reduced freezing levels during retrieval test compared to WT (WT: n = 9, KI: n = 9; two-way ANOVA, genotype x condition, *F*_1,32_ = 7.06, *p* < 0.05, effect of genotype, *F*_1,32_ = 13.36, *p* < 0.001, effect of condition, *F*_1,32_ = 44.73, *p* < 0.0001; Bonferroni posttests ***p < 0.001). (**d**) Correct arm choice rate showed significant impairment in short-term memory in *Lsd1* KI mice (WT: n = 11; KI: n = 8; two-way RM ANOVA, genotype x time, *F*_4,68_ = 1.83, *p* = 0.133, effect of genotype, *F*_1,68_ = 7.53, *p < 0.05, effect of time, *F*_4,68_ = 0.37, *p* = 0.826; Bonferroni posttests, WT vs KI at day 4, *p < 0.05). (**e**) Freezing levels of WT (black) and *Lsd1* KI (red) mice before (pretraining) and 24 h after (retrieval) cued fear conditioning. *Lsd1* KI mice showed comparable freezing levels during retrieval test to WT (WT: n = 8, KI: n = 9; two-way ANOVA, genotype x condition, *F*_1,30_ = 1.94, *p* = 0.174, effect of genotype, *F*_1,30_ = 2.08, *p* = 0.160, effect of condition, *F*_1,30_ = 40.15, *p* < 0.0001; Bonferroni posttests, ns: not significant). All graphs were plotted as mean ± SEM.

To examine whether LSD1 expression is altered in the hippocampus of *Lsd1* KI mice, we performed an immunohistochemical analysis. We found that the expression patterns of LSD1 in the CA1, CA3 and dentate gyrus (DG) areas of hippocampus of *Lsd1* KI mice were mostly similar to that of WT littermates (Fig. 1a).

To examine hippocampus-dependent associative fear memory in *Lsd1* KI mice, we used the contextual fear conditioning paradigm. During training, KI mice showed similar freezing levels compared to age-matched wild type (WT) littermates (Fig. 1b and c). However, when short-term and long-term memories were assessed at 1 h (Fig. 1b) and 24 h (Fig. 1c) after training, respectively, *Lsd1* KI mice showed significantly lower freezing level compared to WT littermates (Fig. 1b and c), suggesting that LSD1 phosphorylation might be required for contextual fear memory formation. To confirm that the short-term memory is impaired in *Lsd1* KI mice, we performed another type of short-term memory test, the T-maze test^21^. During the sample run, mice were forced to choose one of the two target arms by blocking the other arm. In the choice run, mice were rewarded if they chose the unvisited arm. The correct choice rate of WT was increased over the course of the training, while KI mice did not show any significant learning throughout the training (Fig. 1d), demonstrating the impairment of short-term memory in *Lsd1* KI mice. To examine whether the *Lsd1* KI affects the behavior depending on other brain region, we performed auditory fear conditioning test, which is well established as an amygdala-dependent memory test paradigm. During training, KI mice showed similar freezing levels compared to WT littermates (Fig. 1e). When long-term memory was assessed at 24 h after training, *Lsd1* KI mice showed a comparable freezing level to WT littermates (Fig. 1e), indicating that LSD1 S112 phosphorylation might not be required for amygdala-dependent fear memory formation.

**Figure 2:**
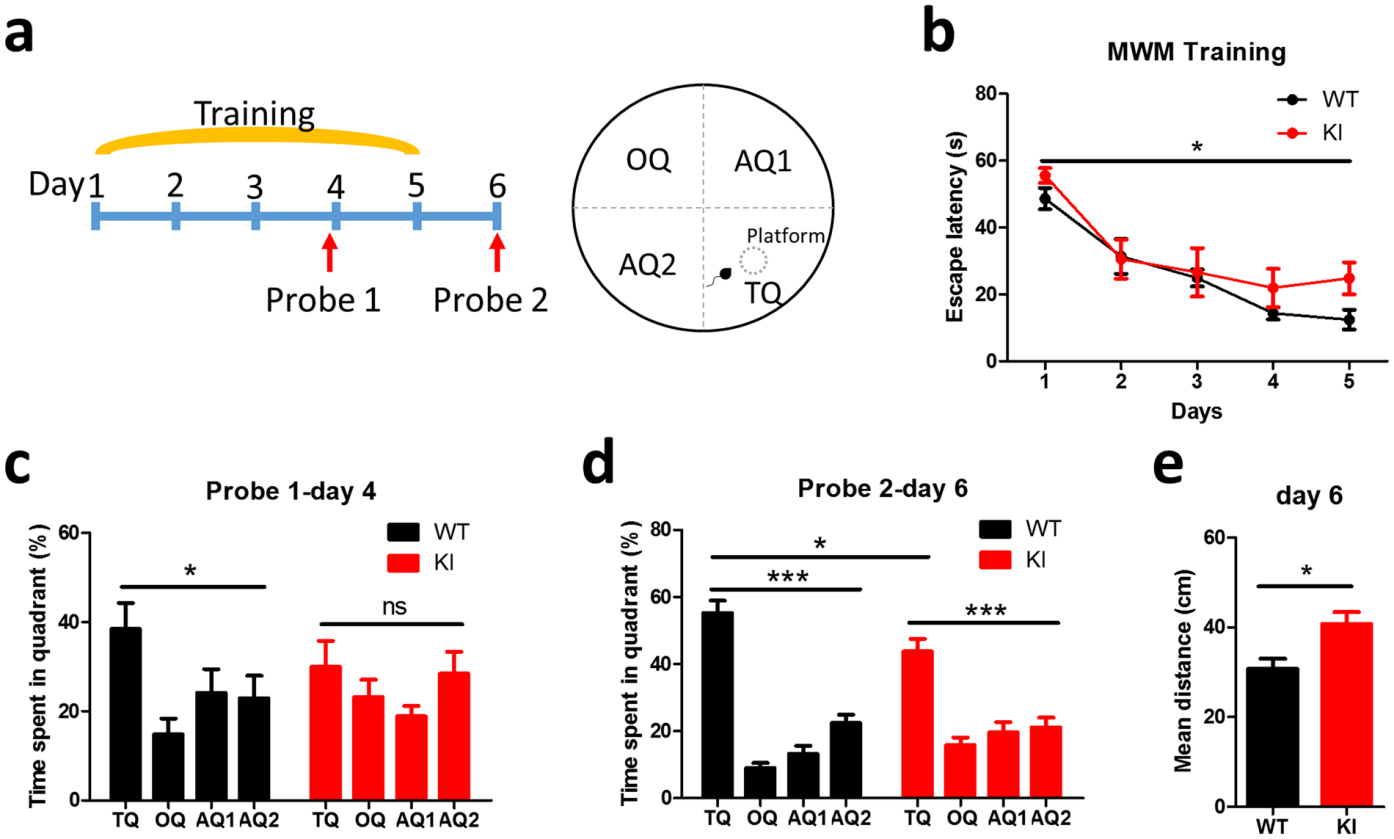
*Lsd1* KI mice show impaired hippocampus-dependent spatial learning and memory. (**a**) Schematic drawings of experimental design and water maze. (**b**) Learning curve during 5 training days of Morris water maze (MWM) task showing the latency for the mice to reach the platform (WT: n = 8, KI: n = 7; two-way ANOVA, genotype x time, *F*_4,65_ = 0.71, *p* = 0.590, effect of genotype, *F*_1,65_ = 4.03, *p < 0.05, effect of time, *F*_4,65_ = 20.16, *p* < 0.0001). (**c**) Time spent in each quadrant during 1-min probe test before training sessions on training day 4 (WT: n = 8, KI: n = 7; one-way ANOVA of WT, *p < 0.05, Bonferroni’s multiple comparison test, TQ vs OQ, *p* < 0.01; one-way ANOVA of KI, ns *p* = 0.307, Bonferroni’s multiple comparison test, TQ vs OQ, ns: not significant). (**d**) Time spent in each quadrant during 1-min probe test 24 h after day 5 training sessions (day 6) (WT: n = 8, KI: n = 7; one-way ANOVA of WT, ***p < 0.0001; one-way ANOVA of KI, ***p < 0.0001; unpaired *t*-test, WT vs KI in TQ, *p < 0.05). TQ: target, OQ: opposite, AQ1: right, AQ2: left quadrant. (**e**) Mean distance from the platform during probe test on day 6 showing significant impairment in spatial memory in *Lsd1* KI mice (WT: n = 8, KI: n = 7; unpaired *t*-test, *p < 0.05). All graphs were plotted as mean ± SEM.

Next, we performed a hippocampus-dependent spatial learning and memory test using the Morris water maze. Both WT and KI mice were trained for 5 consecutive days, with 4 trials per day to locate a hidden platform submerged in a circular pool, filled with opaque water. Two probe trials were given on day 4, before day 4 training, and on day 6, 24 h after the last training (Fig. 2a). In the training trials, the escape latency of *Lsd1* KI mice was significantly longer than that of WT littermates (Fig. 2b), suggesting that spatial learning is impaired in *Lsd1* KI mice. In the first probe trial on day 4, WT mice spent significantly more time in the target quadrant in which a platform was located during training sessions compared to the other quadrants. However, *Lsd1* KI mice spent almost equal time in each quadrant (Fig. 2c). In the second probe trial on day 6, *Lsd1* KI mice spent significantly less time in the target quadrant compared to WT littermates (Fig. 2d), and the mean distance from platform position was significantly longer than that of WT littermates (Fig. 2e), demonstrating that hippocampus-dependent spatial memory is impaired in *Lsd1* KI mice.

To confirm that fear and spatial memory deficits found in *Lsd1* KI mice were not due to changes in anxiety level, we first performed the elevated zero maze (EZM) test^22^ to measure the basal anxiety level in *Lsd1* KI mice (Supple. Figure 1a). Mice naturally prefer to stay in the closed arm of the maze; increased time spent in the open arm indicates lower basal anxiety level of the mouse. *Lsd1* KI mice showed a tendency to spend slightly more time in the open arm compared to WT littermates, although it was not statistically significant (Supple. Figure 1a). Next, we performed the open-field test to measure the anxiety level and locomotor activity in *Lsd1* KI mice. While the time spent in the center was not different between the genotypes (Supple. Figure 1b), the distance moved in 10 min in the open-field was significantly increased in *Lsd1* KI mice (Supple. Figure 1c), suggesting an intrinsic hyperactivity in *Lsd1* KI mice.

### Social recognition memory was impaired in *Lsd1* KI mice

**Figure 3:**
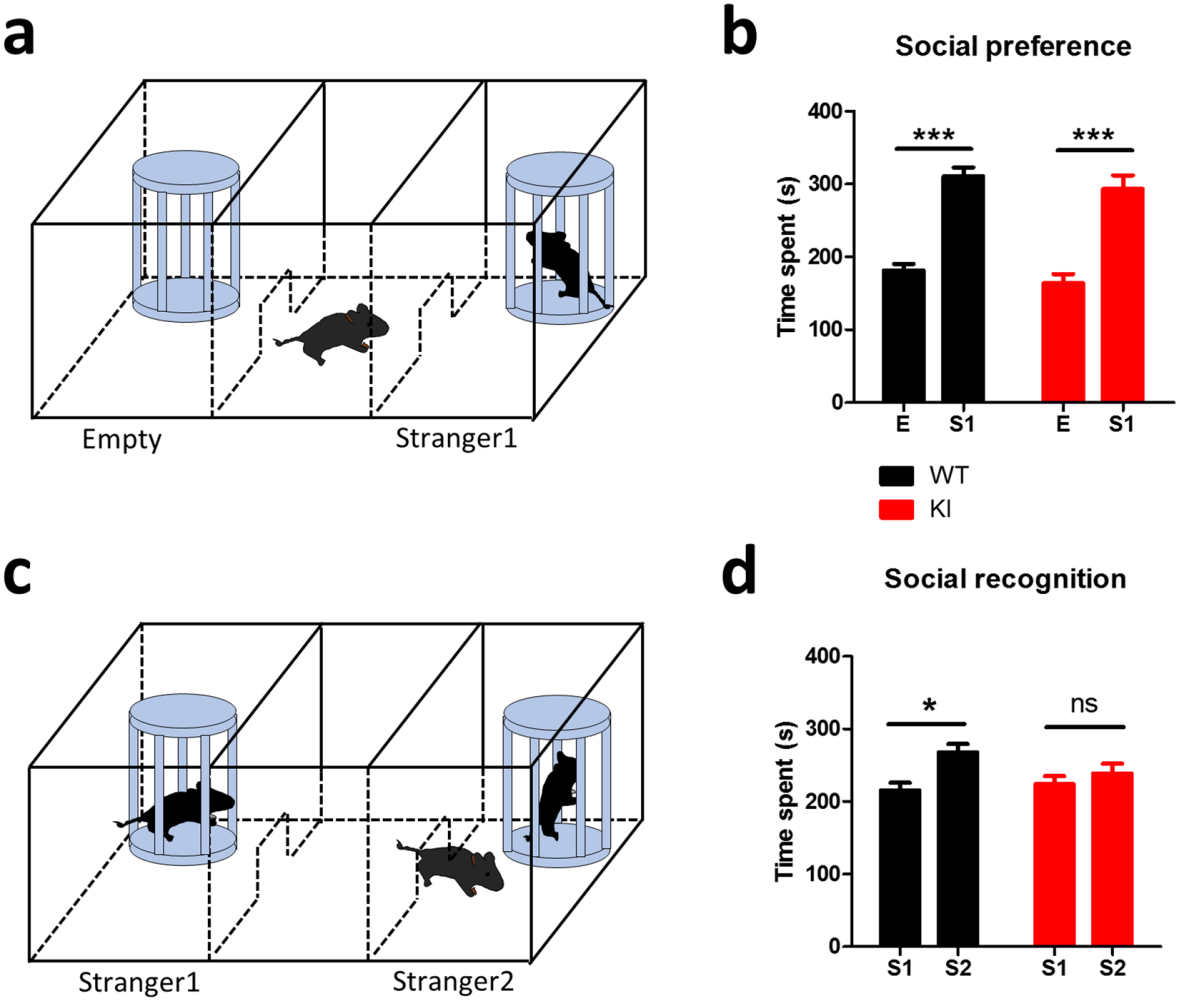
*Lsd1* KI mice show impaired social recognition memory. (**a**) Experimental design for three-chamber test to measure sociability. (**b**) Exploration time for the empty cup (E) and the cup with stranger mouse (S1) of WT and *Lsd1* KI mouse (WT: n = 13, KI: n = 11; two-way ANOVA, genotype x condition, *F*_1,44_ = 0.00, *p* = 1.000, effect of genotype, *F*_1,44_ = 1.78, *p* = 0.189, effect of condition, *F*_1,44_ = 58.21, *p* < 0.0001; Bonferroni posttests, S1 vs E ***p < 0.0001). (**c**) Experimental design for three-chamber test measuring social recognition memory. (**d**) Exploration time for the cup with the familiar mouse (S1) and the cup with the stranger mouse (S2) of WT and *Lsd1* KI mouse (WT: n = 13, KI: n = 11; two-way ANOVA, genotype x condition, *F*_1,44_ = 2.59, *p* = 0.115, effect of genotype, *F*_1,22_ = 0.79, *p* = 0.231; effect of condition, *F*_1,44_ = 8.08, *p* < 0.01; Bonferroni posttests, S2 vs S1 *p < 0.05, ns: not significant). All graphs were plotted as mean ± SEM.

We next performed the three-chamber test to examine the sociability and social memory in *Lsd1* KI mice. Animals with normal sociability show a preference for the cup with a stranger mouse over an empty cup. The *Lsd1* KI mice showed comparable social interaction as WT littermates (Fig. 3a and b). However, during the social recognition memory task which requires normal hippocampal function (Fig. 3c), *Lsd1* KI mice failed to distinguish the novel stranger mouse from the familiar mouse, while WT mice spent significantly more time exploring the novel stranger mouse (Fig. 3d), suggesting social memory deficit in *Lsd1* KI mice.

### *Lsd1* KI mice showed impaired short-term synaptic plasticity

**Figure 4:**
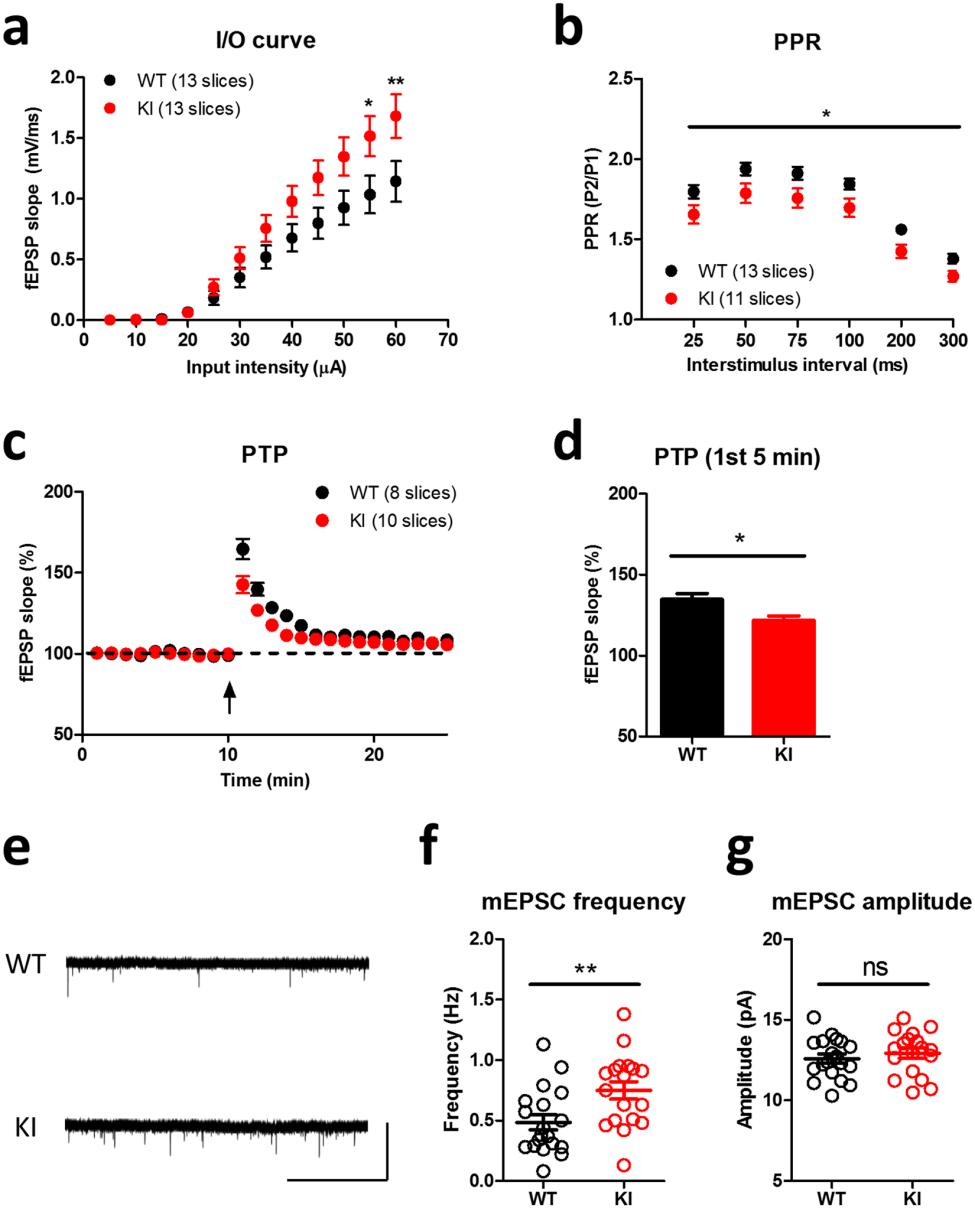
Presynaptic plasticity is changed in *Lsd1* KI mice. (**a**) Input-output relationships at SC-CA1 synapses are significantly increased in *Lsd1* KI mice compared to WT littermates (WT: n = 13, KI: n = 13; two-way RM ANOVA, input intensity x genotype, *F*_11,264_ = 4.46, *p* < 0.0001, effect of input intensity, *F*_11,264_ = 121.54, *p* < 0.0001, effect of genotype, *F*_1,264_ = 3.42, *p* = 0.077; Bonferroni posttests *p < 0.05, **p < 0.01). (**b**) Paired pulse ratio (PPR) was significantly decreased in *Lsd1* KI mice (WT: n = 13, KI: n = 11; two-way RM ANOVA, interstimulus interval x genotype, *F*_5,105_ = 0.36, *p* = 0.874, effect of interstimulus interval, *F*_5,105_ = 245.20, *p* < 0.0001, effect of genotype, *F*_1,21_ = 6.13, *p < 0.05). (**c**) Post-tetanic potentiation (PTP) was significantly reduced in *Lsd1* KI mice (WT, n = 8; KI, n = 10; arrow, 1 × HFS). (**d**) A significant difference in PTP between WT and *Lsd1* KI mice during the first 5 min of recording (WT: 134.7 ± 3.6%, 8 slices from 5 mice, KI: 121.7 ± 2.8%, 10 slices from 5 mice; unpaired *t*-test, *p < 0.05). (**e**) Representative traces of miniature excitatory postsynaptic currents (mEPSCs) recording. Scale bar, vertical: 50 pA; horizontal: 10 sec. (**f**) The frequency of mEPSCs was significantly increased in *Lsd1* KI mice compared with WT littermates (WT: n = 19, KO: n = 18; unpaired *t*-test, **p < 0.01). (**g**) No changes in the amplitude of mEPSCs in *Lsd1* KI mice compared with WT littermates (WT: n = 19, KO: n = 18; unpaired *t*-test, ns: not significant).

Long-term synaptic plasticity such as long-term potentiation (LTP) or long-term depression (LTD) at the hippocampal Shaffer collateral (SC)-CA1 synapses is thought to be a cellular mechanism underlying hippocampus-dependent long-term memory^23–25^. Therefore, we next examined whether the learning and memory deficits observed in *Lsd1* KI mice is correlated with deficits in synaptic plasticity in the hippocampal CA1 region. First, we assessed the basal synaptic transmission of SC-CA1 synapses by examining the input-output relationship and paired-pulse ratio (PPR) using extracellular field recording. *Lsd1* KI mice showed increased input-output relationship at individual stimulus intensity (Fig. 4a), suggesting an enhanced synaptic transmission. Alteration in PPR is a form of presynaptic plasticity^26^, which reflects changes in the release probability of synaptic vesicles at the presynaptic terminals^27^. PPR was significantly decreased in the hippocampal slices of *Lsd1* KI mice compared to WT littermates (Fig. 4b), suggesting that a higher probability of presynaptic neurotransmitter release in *Lsd1* KI mice. To further explore the enhancement of neurotransmitter release probability in the hippocampal synapses of *Lsd1* KI mice, post-tetanic potentiation (PTP) was analyzed using a protocol composed of a single train of tetanic stimulation (100 Hz/s) in the presence of D-APV (D(-)-2-amino-5-phosphonovaleric acid) (50 μΜ) to block NMDA receptor-dependent postsynaptic modifications^28, 29^. PTP is an increase in the neurotransmitter release on a minute time scale after high-frequency stimulation, during which residual Ca^2+^ accumulates in the presynaptic terminal and in turn could activate PKC to enhance neurotransmitter release^30, 31^. PTP was significantly reduced in the hippocampal slices of *Lsd1* KI mice compared to WT littermates (Fig. 3c and d), suggesting that hippocampal presynaptic functions associated with short-term synaptic plasticity are altered in *Lsd1* KI mice. Consistent with the above results, the frequency of miniature excitatory postsynaptic current (mEPSC) of the CA1 neurons in *Lsd1* KI mice was significantly higher than that in WT littermates, while the amplitude was not different between the genotypes (Fig. 4e and f), confirming the increased probability of neurotransmitter release in *Lsd1* KI mice.

**Figure 5:**
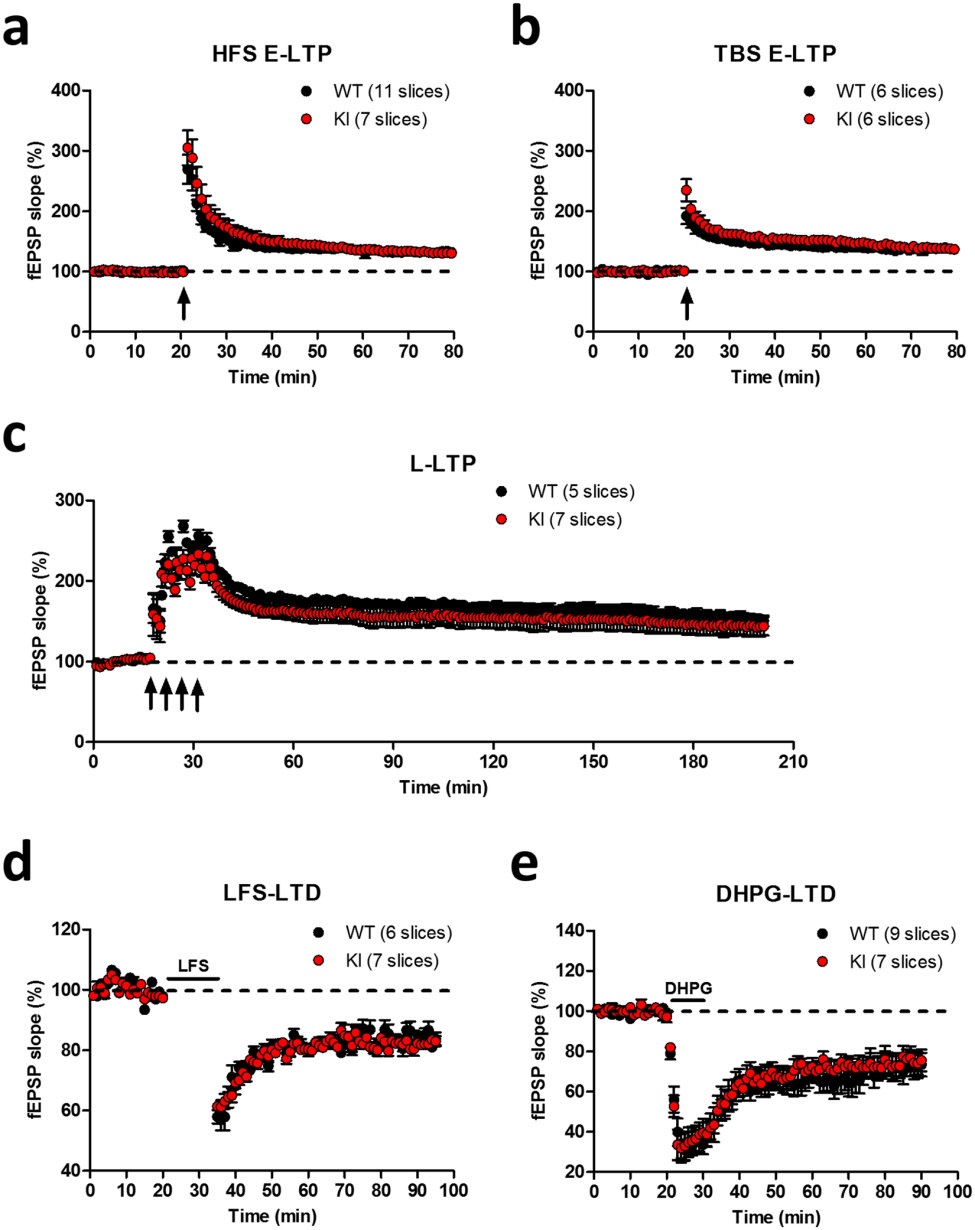
Long-term synaptic plasticity is not changed in *Lsd1* KI mice. (**a**) High frequency stimulation (HFS)-induced early LTP (E-LTP) at SC-CA1 synapses showed no difference in WT and *Lsd1* KI mice (WT: n = 11, KI: n = 7). (**b**) Theta burst stimulation (TBS)-induced E-LTP at SC-CA1 synapses showed no difference in WT and *Lsd1* KI mice (WT: n = 6, KI: n = 6). (**c**) Four trains of high frequency stimulation-induced late-LTP (L-LTP) was not different between WT and *Lsd1* KI mice (WT: n = 5, KI: n = 7). (**d**) NMDA-R-dependent LTD induced by low frequency stimulation (LFS) at SC-CA1 synapses in WT and *Lsd1* KI mice were comparable (WT: n = 6, KI: n = 7). (**e**) DHPG-induced mGluR-LTD at SC-CA1 synapses showed no difference between WT and *Lsd1* KI mice (WT: n = 9, KI: n = 7).

Next, we examined whether long-term synaptic plasticity is altered in *Lsd1* KI mice. Contrary to our expectation, we did not observe any impairment in both high frequency stimulation (HFS)- (Fig. 5a) and theta burst stimulation (TBS)-induced early LTP (Fig. 5b) in *Lsd1* KI mice compared to WT littermates. Late LTP (L-LTP) is a form of synaptic plasticity that is dependent on *de novo* protein synthesis and is considered a mechanism for long-lasting memory^32, 33^. We induced L-LTP in the hippocampal slices by delivering four pulses of high frequency tetanus in 5 min intervals and found that L-LTP also was not different between the genotypes (Fig. 5c). As there were no changes in LTPs in *Lsd1* KI mice, we examined the depressive long-term synaptic plasticity. Both NMDA receptor-dependent LTD induced by low frequency stimulation (LFS) (Fig. 5d) and mGluR-dependent LTD induced by DHPG ((R,S)-3,5-Dihydroxyphenylglycine) treatment (Fig. 5e) were not changed in *Lsd1* KI mice compared to WT littermates.

### Expressions of presynaptic function-related genes were increased in *Lsd1* KI mice

**Figure 6:**
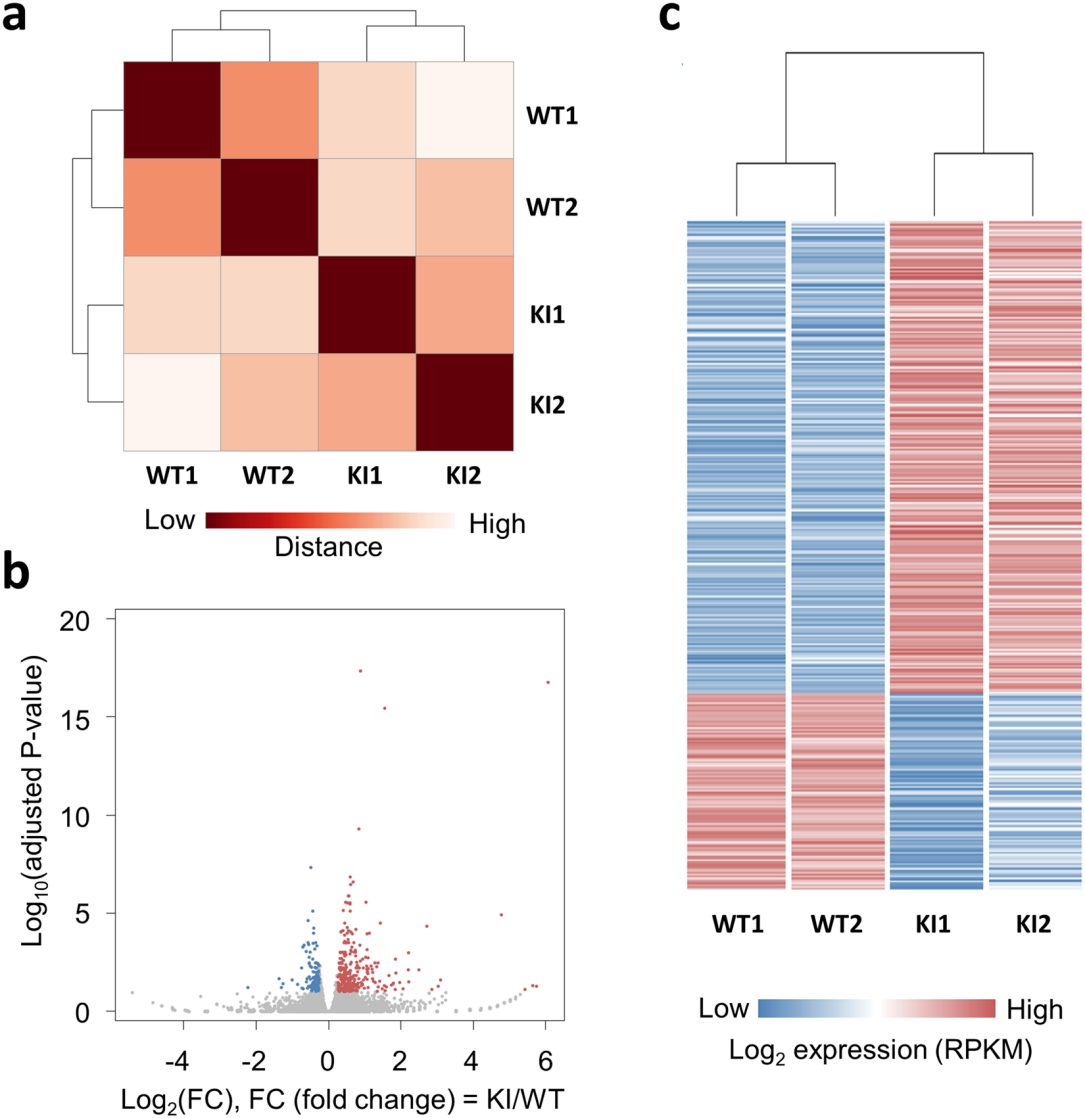
RNA-seq differential gene expression analysis in the hippocampus of *Lsd1* WT and KI mice. (**a**) An expression heatmap of sample-to-sample distances on the matrix using the whole gene expression profiles among two groups (WT and KI) and their replicates (1 and 2). (**b**) Volcano plots show the differentially expressed genes (DEGs) as red (upregulated) and blue (downregulated) dots. The x-axis represents the log2-transformed gene expression in *Lsd1* KI mice divided by that in WT mice. The y-axis is the adjusted *p* value (−log10-transformed) by Benjamini-Hochberg correction. (**c**) A heatmap of expression levels (log2RPKM; reads per kb of exon per million mapped reads) of DEGs in two groups and their replicates.

**Figure 7:**
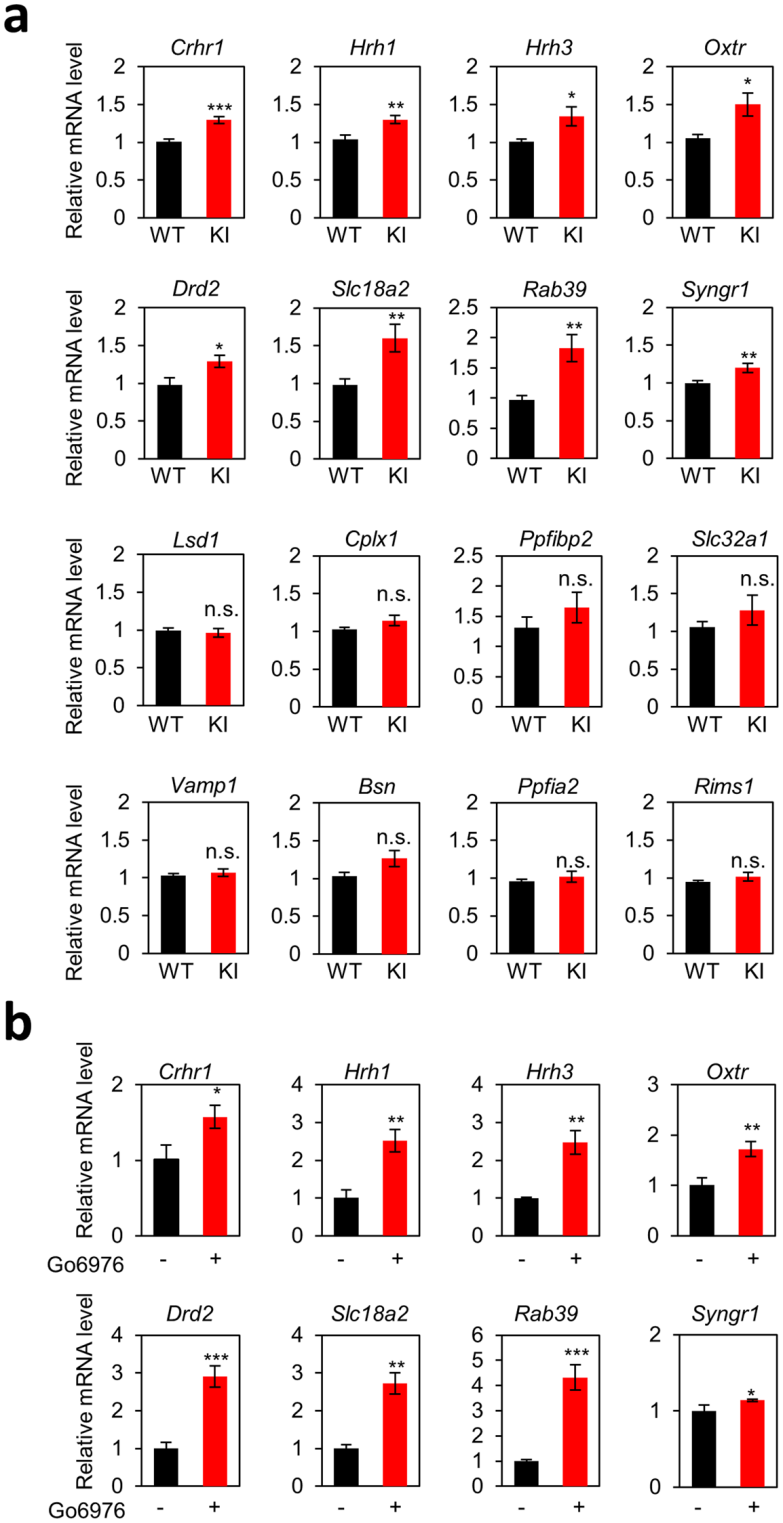
Quantitative RT-PCR analysis of differential gene expression in the hippocampal tissues and cultures. (**a**) Quantitative RT-PCR analysis of differential gene expression showed in RNA-seq analysis in the hippocampus of *Lsd1* WT and KI mice (WT: n = 9, KI: n = 9; unpaired *t*-test, *p < 0.05, **p < 0.01, ***p < 0.001, ns: not significant). Data are expressed as mean ± SEM. (**b**) Quantitative RT-PCR analysis of the changes in gene expression in mouse hippocampal cultures after treatment with Go6976 (100 nM, 8 h), a PKCα inhibitor. (n = 3; unpaired *t*-test, *p < 0.05, **p < 0.01, ***p < 0.001) Data are expressed as mean ± SD.

To gain insight into the molecular basis of the alteration in synaptic transmission, presynaptic plasticity and learning and memory deficits in *Lsd1* KI mice, we performed mRNA sequencing (RNA-seq) analysis and subsequently quantitative RT-PCR (qRT-PCR) analysis from the hippocampus of *Lsd1* KI and control WT littermates. The sample distance measurements among the two groups (WT and KI) and replicates (1 and 2) using the whole gene expression profiles showed that two groups were separated (Fig. 6a). The differential gene expression analysis yielded 381 differentially expressed genes, out of which 271 upregulated and 110 were downregulated (Fig. 6b and c, Supplementary Tables 1 and 2). To get an insight into which transcription factors cooperate with LSD1 to activate or repress the target genes, we analyzed the putative promoters for the differentially expressed genes using Enrichr (http://amp.pharm.mssm.edu/Enrichr/)^34^. Interestingly, we found REST as the major putative transcription factor for the upregulated genes in *Lsd1* KI, whereas we could not find the putative transcription factor for the downregulated genes in *Lsd1* KI (data not shown). REST is a well-known transcription factor that cooperates with LSD1^35, 36^. We next investigated the differentially expressed genes with a focus on presynaptic or postsynaptic plasticity and memory. Most postsynaptic genes such as Calcium/calmodulin-dependent protein kinase II (CaMKII; *Camk2a, Camk2b, Camk2d, and Camk2g*), Postsynaptic density protein-95 (PSD-95; *Dlg4*), Shank (*Shank1, Shank2, and Shank3*), SynGAP (*Syngap1*), AMPA-R (*Gria1*), Homer (*Homer1, Homer2 and Homer3*), NMDA-R (*Grin1, Grin2a, Grin2b, Grin2c, Grin2d, Grin3a, and Grin3b*), Neurolign (*Nlgn1, Nlgn2 and Nlgn3*), mGluR(*Grm1, Grm2, Grm3, Grm4 and Grm5*), GKAP (*Dlgap1, Dlgap2, Dlgap3, Dlgap4 and Dlgap5*), Spine-associated RapGAP (SPAR; *Sipa1l1, Sipa1l2, and Sipa1l3*), nNOS (*Nos1, Nos2 and Nos3*) and GRIP (*Grip1*) showed no changes in expression between *Lsd1* WT and KI. However, several presynaptic function-related genes such as Histamine receptor H1 (H1R; *Hrh1*), Histamine receptor H3 (H3R; *Hrh3*), Dopamine D2 receptor (D2R; *Drd2*), and Vesicular monoamine transporter 2 (VMAT2; *Slc18a2*) were upregulated in KI (Supplementary Table 3). To examine the effect of LSD1 phosphorylation on the presynaptic plasticity- and memory-related gene expression in *Lsd1* KI mice, we expanded and selected the 15 presynaptic function- and memory-related genes (*Crhr1, Hrh1, Hrh3, Oxt, Drd2*, *Slc18a2* (*VMAT2*), *Rab39*, *Syngr1*, *Cplx1*, *Ppfibp2* (*Liprin-b-2*), *Slc32a1* (*VGAT*), *Vamp1*, *Bsn*, *Ppfia2* (*Liprin-a-2*), and *Rims1*) together with *Kdma1* (*Lsd1*) as a control for qRT-PCR verification. The results using total RNA from the hippocampal tissues showed that the increased expressions of *Crhr1, Hrh1, Hrh3, Oxtr, Drd2*, *Slc18a2* (*VMAT2*), *Rab39*, and *Syngr1* genes were consistent with that we measured using RNA-Seq analyses (Fig. 7a). However, the expression level of *Bsn, Ppfia2 (Liprin-a-2)*, and *Rims1* was not significantly different between the genotypes in qRT-PCR analysis (Fig. 7a).

Since LSD1 phosphorylation by PKCα did not occur in *Lsd1* KI mice, we examined whether blocking PKCα activity had similar effects on the expression of presynaptic function-related genes as in *Lsd1* KI mice. It was previously shown that treatment with Go6976, a PKC inhibitor, abrogated PKCα-dependent phosphorylation of LSD1 on S112 site^18^. We treated cultured primary hippocampal neurons with Go6976 (100 nM, for 8 h) and examined the mRNA levels of presynaptic function-related genes. As expected, Go6976 treatment increased the mRNA levels of *Crhr1, Hrh1, Hrh3, Oxtr, Drd2*, *Slc18a2* (*VMAT2*), *Rab39*, and *Syngr1* genes compared to non-treated control group. These data were consistent with the RNA-seq and qRT-PCR analyses in the hippocampal tissues obtained from WT or *Lsd1* KI mice (Fig. 7b). Together, these data indicate that LSD1 phosphorylation by PKCα is required for presynaptic plasticity- and memory-related gene regulation.

### Interaction of LSD1n-SA mutant with HDAC1 was decreased in neuronal cells

**Figure 8:**
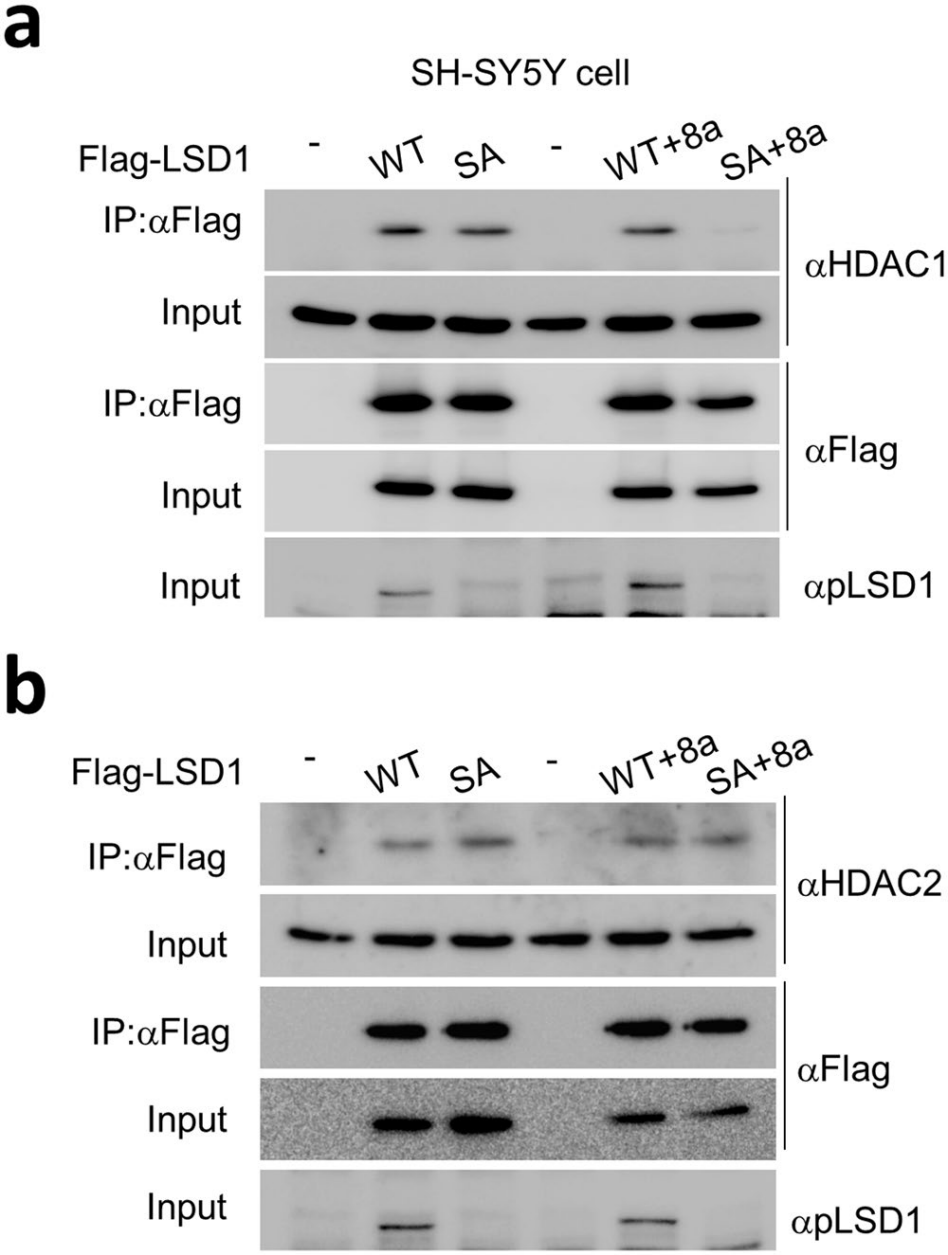
LSD1n-SA mutant show impaired interaction with HDAC1. (**a** and **b**) Co-immunoprecipitation assay of conventional LSD1-WT, -SA mutant and neuron-specific LSD1n-WT, -SA mutant with HDAC1 (**a**) or HDAC2 (**b**) in SH-5YSY cells. Full blot images were included in the Supplementary Information file.

To examine whether the phosphorylation-defective LSD1 mutant (LSD1-SA) affects its interaction with co-repressors, we performed the co-immunoprecipitation assay in SH-SY5Y cells, human neuronal cells derived from neuroblastoma. According to a recent study, SH-SY5Y cells express both the neuron-specific LSD isoform (LSD1n, including 8a exon) and conventional/canonical LSD1 form (without 8a exon), suggesting that these cells can mimic *in vivo* neuronal cells^37^. It has been reported that LSD1n, a dominant-negative neuron-specific splicing variant of LSD1, is critical for proper neurite morphogenesis, learning and memory, and modulation of emotional behavior^15, 38, 39^. We compared the binding affinity of HDAC1 (Fig. 8a) or HDAC2 (Fig. 8b) to conventional LSD1- or LSD1n-WT or -SA mutant to examine whether HDAC1/2 interaction with LSD1 is S112 phosphorylation-dependent. Both conventional LSD1-WT and LSD1-SA showed comparable binding to HDAC1, whereas the interaction between LSD1n-SA mutant and HDAC1 was significantly attenuated compared to LSD1n-WT (Fig. 8a). HDAC2 showed comparable binding to all types of LSD1 (Fig. 8b). These results suggest that phosphorylation of LSD1n isoform modulates the transcription of presynaptic genes through its binding with HDAC1 in neuron.

## Discussion

In this study, we found that *Lsd1* ^SA/SA^ KI mice showed deficits in hippocampus-dependent short-term and long-term fear memory and spatial memory. In addition, *Lsd1* KI mice showed impaired social recognition memory, which is also dependent on the hippocampal function. Presynaptic plasticity such as post-tetanic potentiation (PTP) and paired pulse ratio (PPR) was reduced in *Lsd1* KI mice, whereas no changes in long-term synaptic plasticity, such as LTP and LTD, were observed. Consistent with this, the expression levels of presynaptic plasticity- and memory-related genes were altered in *Lsd1* KI mice.

*Lsd1* KI mice showed clear deficits in long-term contextual fear and spatial memory (Figs 1 and 2). It has been suggested that LSD1 might be required for memory consolidation^14, 40^. It has been also reported that epigenetic modification of histone lysine methylation on H3K9 by LSD1 is involved in gene expression changes necessary for fear memory consolidation and is regulated by NMDA receptor-ERK signaling^12, 13^. Since LSD1 S112 phosphorylation site is not located within the amine oxidase region of LSD1, which is crucial for enzymatic activity, LSD1 phosphorylation status does not affect its intrinsic histone demethylase activity^18^. Here, we focused on the role of LSD1 phosphorylation in hippocampus-dependent learning and memory using *Lsd1* KI mice. Genome-wide RNA-seq analysis of hippocampal tissues from WT and *Lsd1* KI mice revealed that LSD1 phosphorylation is required for transcriptional repression of a wide range of memory- and presynaptic plasticity-related genes. We also observed that Go6976 treatment in cultured primary hippocampal neurons upregulated the expression of the same target genes. To regulate the memory- and presynaptic plasticity-related genes, LSD1n showed altered binding affinity with HDAC1 depending on its phosphorylation status. Previously, we reported that LSD1 phosphorylation induces the interaction with CLOCK:BMAL1 transcription factor, activating E-box mediated transcription to regulate circadian rhythmicity^18^. In addition, LSD1 forms various protein complexes with several transcription factors or cofactors depending on the physiological conditions. LSD1 represses the transcription of neuronal genes with CoREST-HDAC1/2 corepressor complex in non-neuronal cells^41^. Further, LSD1 interacts with androgen receptor (AR) or estrogen receptor (ER) and promotes AR- or ER-dependent transcription in prostate and breast cancer cells, respectively^3, 42^. In this study, we used only male mice and could not examine whether there is any sex-dependent difference in the memory, synaptic plasticity and gene expression between WT and *Lsd1* KI mice.

Since LSD1 forms various complexes with several transcription factors as a corepressor or coactivator, it is possible that phosphorylated LSD1 would interact with the transcriptional repressor complex to suppress the memory- and presynaptic plasticity-related genes, which are important for memory consolidation. Treatment with Go6976 might release LSD1 from the transcriptional repressor complex and lead to the upregulation of LSD1 phosphorylation-dependent target genes. Consistently, we found that the phosphorylation of LSD1n is critical for its binding to HDAC1 in neuronal cells (Fig. 8). However, how and why the interaction with HDAC1 is influenced by the phosphorylation state of LSDn isoform alone needs further investigation.

Previous study showed that *Lsd1* KI mice had altered circadian rhythms in locomotor behavior with reduced rhythmic expression of the core clock genes^18^. Many reports showed that the perturbation of circadian clock has profound effects on mood and memory^19, 20^. *Bmal1*, a clock timing gene, knockout (KO) mice exhibit impaired contextual fear and spatial memory, without impairment in anxiety-related behaviors and LTP at the SC-CA1 synapse^19, 20^. In addition, MEK-ERK and cAMP signal transduction pathways in the hippocampus during contextual fear conditioning are absent in *Bmal1* KO, suggesting that BMAL1 is required for critical signaling events in the hippocampus for memory formation^19^. It would be interesting to examine whether there are any changes in the activity of MAPK pathway or cAMP pathway in *Lsd1* KI mice according to the circadian rhythm.

*Lsd1* KI mice also showed short-term contextual fear and spatial reference memory deficits (Fig. 1). Consistent with these results, PTP and PPR were decreased; however, the frequency of mEPSC was increased (Fig. 4), strongly suggest that the presynaptic neurotransmitter release is abnormally increased and subsequently impairs short-term plasticity in *Lsd1* KI mice. Although long-term synaptic plasticity is known as a major cellular mechanism for learning and memory in animals, many reports suggest that short-term synaptic plasticity is also involved in the regulation of associative learning and memory. For example, Silva *et al*.^43^ found impaired learning in several genetically modified mouse lines, including heterozygous CaMKIIα deletion (CaMKIIα^+/−^) and Synapsin II KO mice, which showed normal CA1 LTP but impairment in several forms of short-term plasticity. CaMKIIα heterozygous mice show lower PPR and enhanced PTP; however, Synapsin II KO mice show no change in PPR but lower PTP^43^. In addition, regulating synaptic membrane exocytosis protein 1 alpha (RIM1α), a presynaptic protein that is required for maintenance of normal neurotransmitter release^44^ and long-term presynaptic potentiation^45^, KO mice that are impaired in long-term fear memory also show impaired short-term plasticity, enhanced PPR and lower PTP, but no changes in CA1 LTP^46^. Moreover, it was reported recently that the Synapses of amphids defective (SAD)-B KO mice show enhanced PPR and decreased mEPSC frequency with no changes in PTP and LTP in the CA1 region but show long-term fear memory deficit^29^. Although there is no conclusive explanation regarding how changes in these different forms of presynaptic plasticity affect cognition, it was suggested that the combinations of several presynaptic abnormalities affect impaired associative memory formation^29, 46^.

In conclusion, our results demonstrate the critical role of LSD1 phosphorylation by PKCα on learning and memory in the adult mouse brain.

## Methods

### Mice

Age-matched (8–15 weeks old) male *Lsd1* knock-in (KI) and wild type (WT) littermates were used for the behavioral experiments. Mice were housed in a 12 h light/dark cycle in standard laboratory cages, and the behavioral experiments were performed during the light phase of the cycle. Food and water were provided *ad libitum*. The Institutional Animal Care and Use Committee (IACUC) of Seoul National University approved the animal protocols. All experiments were performed in accordance with their guidelines and regulations.

### Immunohistochemistry

Age-matched WT and *Lsd1* KI mice were perfused with 4% paraformaldehyde (PFA) in PBS. The brains were removed and kept in 4% PFA overnight at 4 °C. Brain sections (40 μm thickness) were collected by sectioning with a cryostat and incubated in a blocking solution (2% goat serum, 0.2% Triton X-100 in PBS) for 1 h and then with the blocking solution containing an LSD1 antibody (1:500, abcam) overnight at 4 °C. Sections were then incubated with the blocking solution containing anti-rabbit Alexa Fluor 555 IgG (1:400, Invitrogen) for 2 h at room temperature. Fluorescent images were captured using a fluorescent microscope (IX51, Olympus).

### Behavioral tests

#### Elevated zero maze (EZM) test

Mice were placed in the center of one of the closed arms of the maze, and their movement was tracked for 5 min using a tracking program (EthoVision 9.0, Noldus) under bright light. The maze, made of white Plexiglas, was round-shaped (50 cm diameter, 5 cm width), and the platform was elevated 65 cm above ground level. Two closed arm regions had 20 cm walls on both sides.

#### Open-field test

Mice were placed in the center of a square opaque white box (40 × 40 × 40 cm) under dim light, and the movement of each mouse in the central (within a 20 × 20 cm) and peripheral areas of the box was tracked using a tracking program (EthoVision 9.0, Noldus) for 10 min.

#### T-maze test

T-maze test was performed as previously described^21, 47, 48^ with some modification. Briefly, mice were group-housed and food-deprived by feeding them 80~85% of average *ad libitum* daily intake. Mice were handled for 3 min per day for 3 consecutive days. For habituation, mice were exposed to the maze (long arm = 41 cm × 9 cm × 10 cm, short arms = 30 cm × 9 cm × 10 cm, start box = 8 cm × 8 cm × 10 cm) for 15 min on 2 consecutive days with reward (50% condensed milk diluted with saline). Mice were tested on 4 trials per day. Each trial comprised two runs: a forced run and a choice run. The forced run arm and choice run arm was randomly switched. All tasks were performed under dim light.

#### Contextual fear conditioning (CFC) test

After 3-min handling for 4-consecutive days, mice were trained for contextual fear conditioning. Mice were placed in a conditioning chamber and presented with a single electrical foot shock (0.6 mA for 2 sec). Then, mice were returned to the dam. For fear memory test, mice were placed in the same chamber and exposed to the same context for 3 min at 1 h or 24 h after training. Freezing (immobile posture except for respiration) level was measured automatically using a computer program (Freeze Frame, Coulbourn Instruments).

#### Cued fear conditioning test

Mice were handled for 3 min for 4-consecutive days. On conditioning day, mice were placed in a conditioning chamber and a tone (3 kHz, 80 dB) was delivered for 30 sec at 3 min and 5 min, which was terminated by an electrical foot shock (0.7 mA for 2 sec). Twenty four hours after the conditioning, the mice were placed in a modified chamber having different context for 3 min and then the tone was presented for another 1 min. Percent time of freezing was automatically measured using the FreezeFrame2 software (Coulbourn Instruments).

#### Morris water maze (MWM) test

MWM test was performed as previously described^49^. Mice were handled for 3 min at the same time for 3 consecutive days before performing the test. For training, mice were put into a white-opaque water (20~22 °C)-filled tank (140 cm diameter, 100 cm height) placed in a room with multiple spatial cues. The tank was divided into 4 virtual quadrants, and a 10 cm diameter-platform was placed at the center of a quadrant (TQ). On the training days, mice were randomly released at the edge of the maze facing the inner wall of the tank and trained to reach the platform for 60 s. If the mice failed to reach the platform within 60 s, they were guided to or placed on the platform for 10 s. When the mice successfully reached the platform and stayed on the platform for more than 1 s, they were rescued from the maze after 10 s. Mice were trained with 4 trials per day, and the trial interval was 2 min. Probe tests were performed in the same condition with training trials except for the absence of the platform, and the mice were tracked for 60 s with a tracking program (EthoVision 9.0, Noldus). Probe test 1 was performed on training day 4 before training trials began. On day 6, probe test 2 was performed 24 h after the last training trial on day 5.

#### Three-chamber test

Three-chamber test was performed as previously described^49^. Stranger mice were handled for 3 min and then habituated in a wire cage placed in the 3-chamber apparatus for 5–10 min for 4 consecutive days. When the handling was over, the test mouse was habituated to the 3-chamber apparatus for 10 min with the doors opened. When the habituation was over, the test mouse was guided to the center chamber, and the doors were closed. A wired cup with a stranger mouse (stranger 1) and an empty cup was introduced into the other two chambers, and then the doors were opened for sociability test. The movement of the test mouse was tracked for 10 min with a tracking program (EthoVision 9.0, Noldus). When the sociability test session was over, the test mouse was guided to the center chamber, and the doors were closed. Another stranger mouse (stranger 2) was introduced into the empty cup for a social recognition test. The doors were opened, and the movement of the test mouse was tracked for another 10 min. For each set of experiments, the orientation of the two wired cups containing stranger 1 or stranger 2 (or empty) was counter-balanced.

#### Electrophysiology

For extracellular field recordings, transverse hippocampal slices (400 μm thick) were prepared from 4~5-week-old mice deeply anesthetized with isoflurane for LTD or 8~12-week-old mice for LTP experiments using a manual tissue chopper and incubated in a recovery chamber for at least 2 h. After the recovery period, the slices were placed in a recording chamber at 25 °C, perfused (1~1.5 mL/min) with oxygenated artificial cerebrospinal fluid (ACSF, 290 Osm) containing (in mM) 124 NaCl, 2.5 KCl, 1 NaH_2_PO_4_, 25 NaHCO_3_, 10 glucose, 2 CaCl_2_, and 2 MgSO_4._ Extracellular field EPSPs (fEPSPs) were recorded from the CA1 area using a glass electrode filled with ACSF (1 MΩ). The Schaffer collateral (SC) pathway was stimulated every 30 s using concentric bipolar electrodes (MCE-100; Kopf Instruments). Field potentials were amplified, low-pass filtered (GeneClamp 500; Axon Instruments), and then digitized (NI PCI-6221; National Instruments) for measurement. Data were monitored, analyzed online, and reanalyzed offline using WinLTP program. For LTP and LTD experiments, after a stable baseline was recorded, high frequency stimulation (100 Hz, 1 s for HFS-LTP), four trains of high frequency stimulation (4 × 100 Hz, 1 s each, 5 min intertrain interval for HFS-L-LTP), low frequency stimulation (1 Hz, 900 stimuli for LFS-LTD), theta burst stimulation (3 × TBS, 1 s each for TBS-LTP), or (R,S)-3,5-Dihydroxyphenylglycine (DHPG) (100 μM for 10 min for DHPG-LTD) was delivered. For post-tetanic potentiation (PTP), the NMDA receptor antagonist D(-)-2-amino-5-phosphonovaleric acid (D-APV, 50 μM, Tocris), was added in ACSF during recording. Whole-cell patch clamp recordings were performed in hippocampal slices (300 μm thick) prepared using a vibratome (VT1200S; Leica). Slices were allowed to recover for at least 1 h in a recovery chamber at room temperature (RT) with ACSF. After recovery, hippocampal tissue after cutting out the CA3 region from the slice was transferred to a recording chamber and maintained at RT with oxygenated ACSF. For experiment that examine miniature excitatory postsynaptic current (mEPSC), the recording pipettes (3~5 MΩ) were filled with an internal solution containing (in mM) 100 Cs-gluconate, 5 NaCl, 10 HEPES, 10 EGTA, 20 TEA-Cl, 3 QX-314, 4 MgATP, and 0.3 Na3GTP (280~300 mOsm, pH adjusted to 7.2 with CsOH). Picrotoxin (100 μM) was added to the ACSF to block the GABA-receptor-mediated currents. To measure mEPSC, tetrodotoxin (1 μM) was added additionally. For the spontaneous inhibitory postsynaptic current (sIPSC) recording, we used the following internal solution (in mM) 145 KCl, 5 NaCl, 10 HEPES, 10 EGTA, 10 QX-314, 4 MgATP, and 0.3 Na3GTP (280~300 mOsm pH adjusted to 7.2 with KOH) in the presence of AP5 (50 μM) and CNQX (100 μM). Hippocampal neurons were voltage-clamped at −70 mV using a Multiclamp 700B (Molecular Devices). Only cells with a change in access resistance <20% were included in the analysis. MiniAnalysis program (Synaptosoft) was used for mEPSC and sIPSC analyses.

### RNA-seq analysis

Total RNA was extracted from mouse hippocampal tissues using TRIzol (Invitrogen). The integrity of the extracted total RNA was analyzed using BioAnalyzer, and the standard Illumina protocol was used to prepare the libraries for RNA-Seq. Using gel electrophoresis, the DNA fragments in the libraries with an insert size of ~300 bp were isolated, amplified, and sequenced using a Illumina HiSeq 2000 sequencer in the paired-end sequencing mode (2 × 151 bp reads). The GSNAP alignment tool (2013–11–27) [PMID: 20147302] was used to align all sequencing raw reads to the mouse genome (mm10) reference sequence. Only uniquely and properly mapped read pairs were used for further analysis. To identify the differentially expressed genes between KI and WT samples, edgeR package [PMID: 19910308] was used. Differentially expressed genes were selected as those with changes of at least 1.5-fold between samples and at a false discovery rate (FDR) cutoff of 10% based on edgeR-adjusted *p* values.

### Quantitative Real-time PCR (qRT-PCR)

Total cellular RNA was extracted using TRIzol (Invitrogen). RNA was reverse-transcribed with oligo (dT) primers and M-MLV Reverse Transcriptase (Enzynomics). The obtained cDNA was mixed with TOPreal™ qPCR 2X PreMIX (SYBR Green, Enzynomics) and gene-specific primers for PCR. The abundance of mRNA was detected using CFX384 Touch^TM^ Real-Time PCR Detection System (Bio-Rad) or ABI 7500 System with SYBR Green. The cycling conditions were 95 °C for 15 min, followed by 40 cycles of 95 °C for 10 s, 60 °C for 15 s, and 72 °C for 30 s. Amplification of β-actin was performed in parallel for each sample and used as an internal control. Primer sequences are listed in Supplementary Table 4.

### Co-immunoprecipitation assay and immunoblot analysis

Cells were briefly rinsed with ice-cold PBS and harvested with PBS. Cells were lysed in IP150 buffer (25 mM Tris–HCl pH 7.8, 1 mM EDTA, 10% glycerol, 150 mM NaCl and 0.1% NP-40) supplemented with protease inhibitors. After preserving 10% input, total cell lysates were rotated with Flag antibody (F3165, Sigma) at 4 °C for 2 h. 30 μl of a 50% slurry of protein G–Sepharose and A-Sepharose in IP150 buffer were then added to the reaction mixtures and incubated for 1 h at 4 °C. After rapid centrifugation, the resulting Sepharose beads were washed four times with IP150 buffer, and boiled for 10 min with addition of 2X sample buffer. Co‐immunoprecipitated proteins were analyzed by SDS–PAGE followed by immunoblotting using anti‐Flag (1:5000, F3165 from Sigma), anti-HDAC1 (1:500, sc-6298 from Santa Cruz), anti‐HDAC2 (1:250, sc-7899 from Santa Cruz) or pLSD1 (1:250, ABE 1462 from Millipore) antibodies diluted in 3% BSA PBS-T for overnight. After washes with PBS-T, the membrane was incubated with the species-appropriated HRP-conjugated secondary antibody (Jackson, 115-035-003, 211-032-171) for 1 hr and then washed with PBS-T. Immuno-labelled proteins were visualized using LumiFlash Ultima Chemiluminescent substrate (Visual Protein) by LAS-4000 mini (Fuji).

### Statistics

We did not employ any statistical methods to pre-determine the animal number or sample size. However, the number of animals or size of samples in this study was similar to those generally accepted in the field. To determine whether the data presented here follow normal distribution, we performed either D’Agostino & Pearson omnibus, Shapiro-Wilk or Kolmogorov-Smirnov normality test (Supplementary Table 5). To analyze the fear conditioning data, two-way analysis of variance (ANOVA) was used (between-group factor: genotype; within-group factor: condition). For the Morris water maze data, we performed two-way repeated measure (RM) ANOVA to analyze escape latency (between-group factor: genotype; within-group factor: time) or one-way ANOVA to measure the quadrant occupancy (% time spent in quadrant) and then followed by Bonferroni posttests to evaluate pair-wise group differences. When two groups were compared, either unpaired two-tailed *t*-test or Mann Whitney test was applied depending on the result of normality test. All the analyses were performed using GraphPad Prism 5.01 or 6.01 program. Data are represented as mean ± standard error of mean, SEM (or standard deviation, SD).

### Data availability

RNA-Seq data were submitted to the NCBI Gene Expression Omnibus (GEO) with ID GSE94018.

## Electronic supplementary material

Supplementary information

Supplementary Table S1

Supplementary Table S2

Supplementary Table S3

Supplementary Table S4

Supplementary Table S5
