# Supplementary material for: PKCα-mediated phosphorylation of LSD1 is required for presynaptic plasticity and hippocampal learning and memory
Source: Sci Rep. 2017 Jul 7;7:4912. doi: 10.1038/s41598-017-05239-7 (PMC5501860; doi:10.1038/s41598-017-05239-7)
Supplement: Supplementary file 1 — Supplementary information [file 41598_2017_5239_MOESM1_ESM.pdf]

PKCα-mediated phosphorylation of LSD1 is required for presynaptic plasticity and hippocampal learning and memory

Chae-Seok Lim[1,*], Hye Jin Nam[2,*], Jaehyun Lee[1], Dongha Kim[2], Ja Eun Choi[1], SukJae Joshua Kang[1], Somi Kim[1], Hyopil Kim[1], Chuljung Kwak[1], Kyu-Won Shim[1], Siyong Kim[1], Hyoung-Gon Ko[1], Ro Un Lee[1], Eun-Hae Jang[1], Juyoun Yoo[1], Jaehoon Shim[1], Md Ariful Islam[1], Yong-Seok Lee[3], Jae-Hyung Lee[4], Sung Hee Baek[2,#], Bong-Kiun Kaang[1,#]

[1]Laboratory of Neurobiology, [2]Laboratory of Molecular and Cellular Genetics, School of Biological Sciences, College of Natural Sciences, Seoul National University, Seoul 08826, Korea

[3]Department of Physiology, Biomedical Sciences, Seoul National University College of Medicine, Seoul 03080, Korea

[4]Department of Life and Nanopharmaceutical Sciences, Department of Maxillofacial Biomedical Engineering, School of Dentistry, Kyung Hee University, Seoul 02447, Korea

[#]Corresponding authors: Sung Hee Baek (sbaek@snu.ac.kr) or Bong-Kiun Kaang (kaang@snu.ac.kr)

Department of Biological Sciences, College of Natural Sciences, Seoul National University, Seoul 08826, Korea

[*]These authors contributed equally to this work.

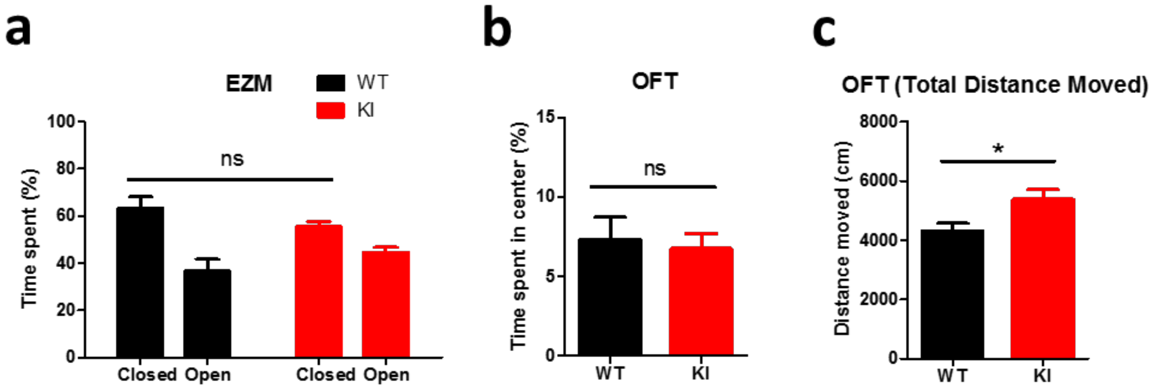

Figure S1. ***Lsd1*** **KI mice show normal basal anxiety and increased locomotion.**

(a) *Lsd1* KI mice showed normal anxiety level in the elevated zero maze (EZM) test (WT: n = 9, KI: n=9; two-way ANOVA, genotype x sector, $F_{1,32} = 4.21$, $p < 0.05$; effect of genotype, $F_{1,32} = 0.00$, $p = 1.000$; effect of sector, $F_{1,32} = 24.57$, $p < 0.0001$; Bonferroni posttests, WT vs KI in closed sector, ns: not-significant) (b-c) In the open-field test, *Lsd1* KI mice showed comparable amount of time spent in the center region (b, unpaired *t*-test, ns: not significant), while locomotor activity was significantly increased compared to WT (c, WT: n = 9, *Lsd1* KI: n = 9; unpaired *t*-test, * $p < 0.05$).

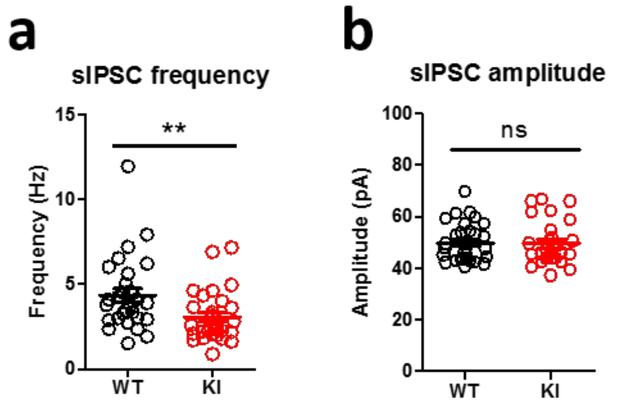

Figure S2. Spontaneous inhibitory postsynaptic currents (sIPSCs) in *Lsd1* KI mice.

Lsd1 KI mice showed decreased sIPSCs frequency (a, WT: n = 30, KI: n = 29; Mann Whitney test, ** $p < 0.01$) but no changes in amplitude (b, WT: n = 30, KI: n = 29; unpaired *t*-test, ns: not significant).

Full blot images for Figure 8a

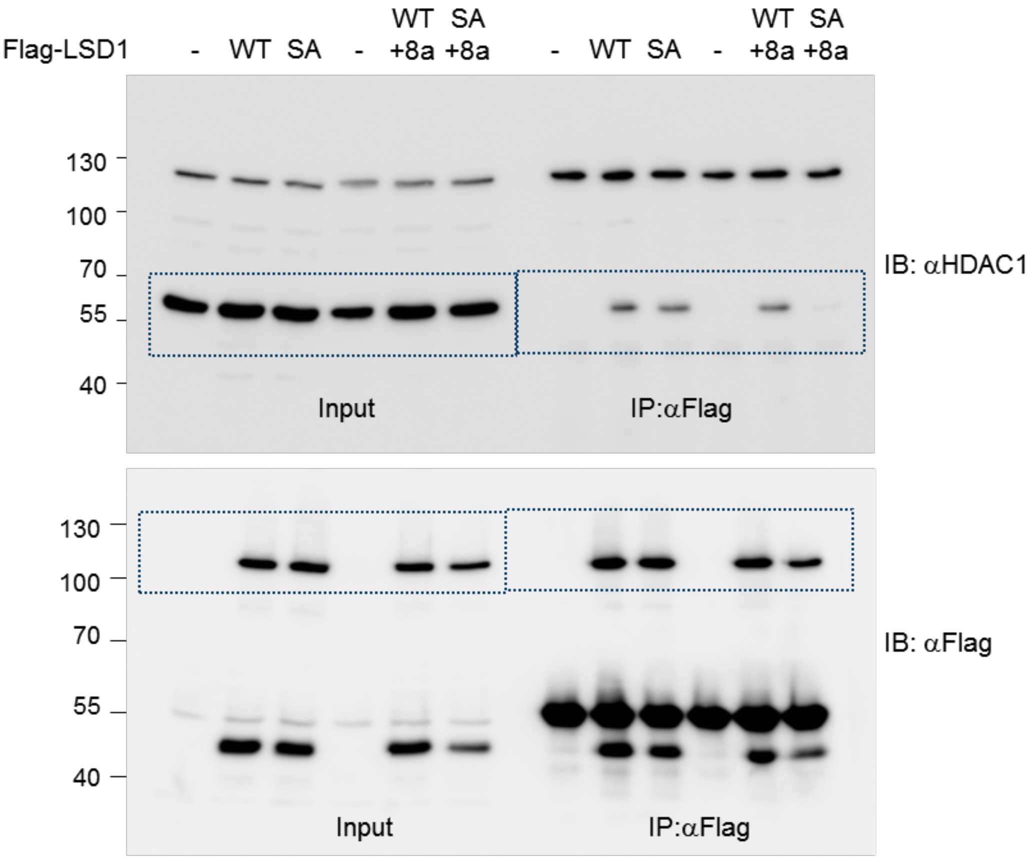

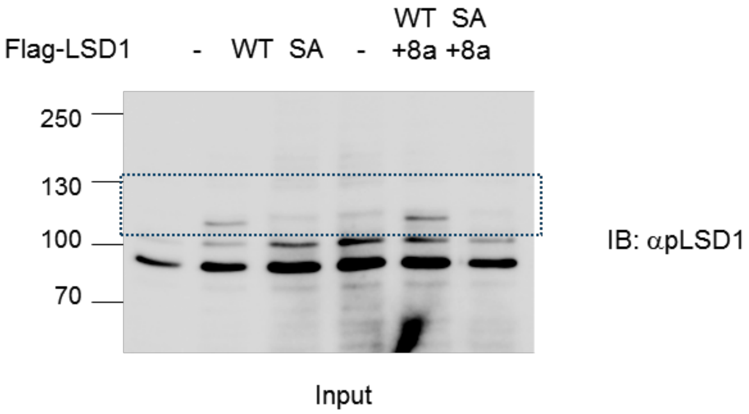

Full blot images for Figure 8b

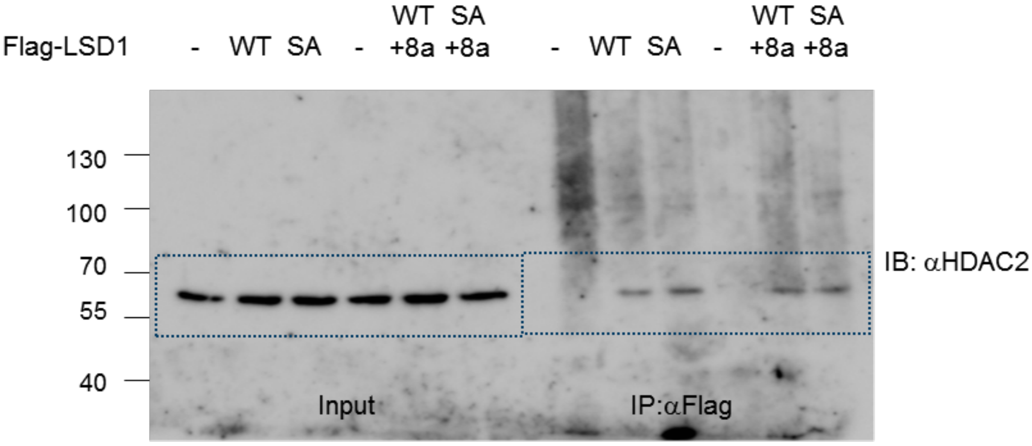

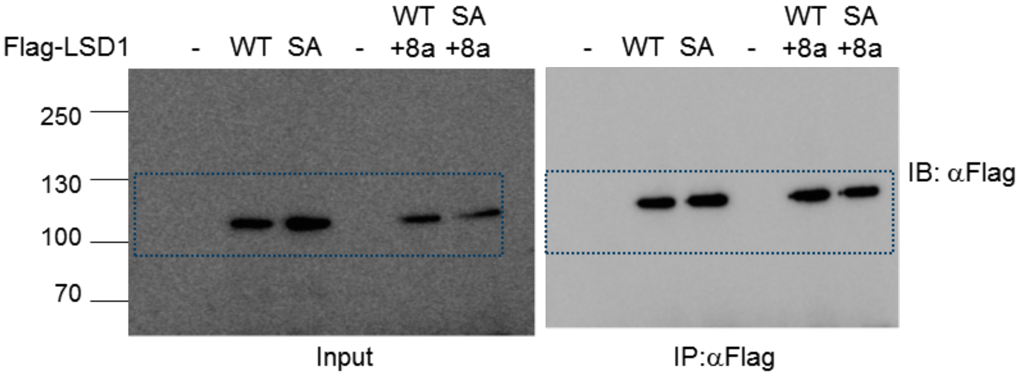

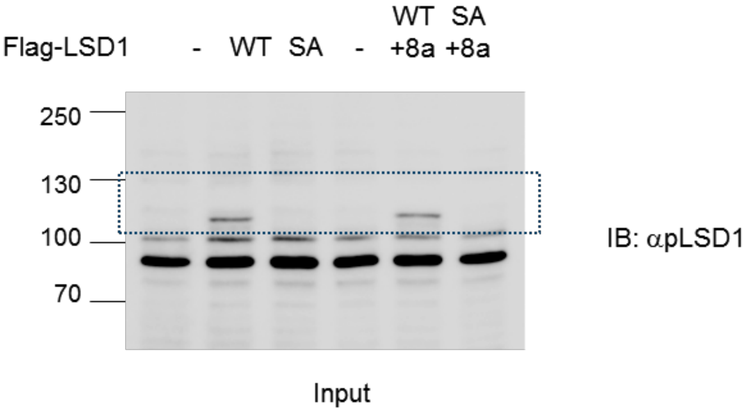

List of Supplementary Tables (Excel Files)

Supplementary Table 1. Upregulated genes in *Lsd1* KI

Supplementary Table 2. Downregulated genes in *Lsd1* KI

Supplementary Table 3. Expression changes in postsynaptic and presynaptic genes

Supplementary Table 4. Primer sequences for qRT-PCR

Supplementary Table 5. Normality test
